# The Moral Obligation to Prioritize Research Into Deep Brain Stimulation Over Brain Lesioning Procedures for Severe Enduring Anorexia Nervosa

**DOI:** 10.3389/fpsyt.2018.00523

**Published:** 2018-10-26

**Authors:** Jonathan Pugh, Jacinta Tan, Tipu Aziz, Rebecca J. Park

**Affiliations:** ^1^The Oxford Uehiro Centre for Practical Ethics, University of Oxford, Oxford, United Kingdom; ^2^Medicine, Swansea University, Swansea, United Kingdom; ^3^Nuffield Department of Clinical Neurosciences, University of Oxford, Oxford, United Kingdom; ^4^Department of Psychiatry, Warneford Hospital, University of Oxford, Oxford Health NHS Foundation Trust, Oxford, United Kingdom

**Keywords:** deep brain stimulation, neurosurgery for psychiatric disease, medical ethics, anorexia nervosa, autonomy

## Abstract

Deep Brain Stimulation is currently being investigated as an experimental treatment for patients suffering from treatment-refractory AN, with an increasing number of case reports and small-scale trials published. Although still at an exploratory and experimental stage, initial results have been promising. Despite the risks associated with an invasive neurosurgical procedure and the long-term implantation of a foreign body, DBS has a number of advantageous features for patients with SE-AN. Stimulation can be fine-tuned to the specific needs of the particular patient, is relatively reversible, and the technique also allows for the crucial issue of investigating and comparing the effects of different neural targets. However, at a time when DBS is emerging as a promising investigational treatment modality for AN, lesioning procedures in psychiatry are having a renaissance. Of concern it has been argued that the two kinds of interventions should instead be understood as rivaling, yet “mutually enriching paradigms” despite the fact that lesioning the brain is irreversible and there is no evidence base for an effective target in AN. We argue that lesioning procedures in AN are unethical at this stage of knowledge and seriously problematic for this patient group, for whom self-control is particularly central to wellbeing. They pose a greater risk of major harms that cannot justify ethical equipoise, despite the apparent superiority in reduced short term surgical harms and lower cost.

Anorexia Nervosa (AN) has the highest mortality rate of all psychiatric disorders (1), and existing treatment modalities have limited effect (2). In this context, there has been burgeoning interest in applying Deep Brain Stimulation (DBS) to the treatment of patients suffering from Severe and Enduring Anorexia Nervosa (SE-AN) (3). Whilst there is some disagreement about the precise diagnostic thresholds for when AN develops into SE-AN (4), here we shall adopt a widely accepted definition of SE-AN according to which a patient suffers from SE-AN if they have suffered from AN for over 7 years, and they have exhausted intensive inpatient and outpatient treatment options (5). SE-AN is thus distinct from other manifestations of AN that have not yet met duration and/or treatment-resistant criteria.

Concurrently, ablative neurosurgery has also been posited as a rivaling treatment for psychiatric disorders. A recent Chinese study by Liu et al. (6) investigated Bilateral Anterior Capsulotomy in the treatment of patients of AN of lesser (>3 years) duration. Notably, participants had not received all alternative treatment modalities recommended by evidence-based guidelines provided by organizations such as the National Institute for Health and Care Excellence [e.g., (7)], as these were not available. For example, participants in the study had only undergone 3 months of psychotherapy, whilst NICE recommends that individual eating-disorder-focused Cognitive Behavioral Therapy (CBT-ED) should typically consist of up to 40 sessions over 40 weeks (7). Accordingly, participants in Liu et al.'s study do not qualify as suffering from SE-AN according to the definition that we adopt here.

In Liu et al.'s study, BMI increased, but as there was no measure of eating disorder psychopathology, it is unclear if this was a consequence of on-going disordered eating. The authors respectfully acknowledged that their patients are different from those deemed treatment refractory in other countries, as there was little available eating disorder expertise in China-and that had this been available these patients might have recovered without surgery. Despite this, and of concern to us, a published follow-up comment on this study suggested that ethical research into DBS and stereotactic ablation for AN can and should proceed in parallel.

Here, we argue in favor of prioritizing research into DBS as a neurosurgical treatment modality for SE-AN, and explain why at this point in our scientific understanding DBS should be prioritized over ablative interventions, and reserved for patients with SE-AN as we have defined it here.

All neurosurgical interventions for SE-AN must currently be considered experimental. Therefore, great care must be taken to protect patients from harm (8). Of course, patients receiving DBS for any indication are unavoidably put at risk of some harm, since they must first undergo an invasive neurosurgical procedure. They must also maintain an implanted foreign device for a long period of time. However, since there are few published reports of DBS in the treatment of SE-AN, it is difficult to fully assess its safety and efficacy in this context. Nonetheless, the feasibility of this approach has been demonstrated (3).

Whilst the risk posed by DBS for movement disorders is generally deemed to be acceptably low, the potential risk of surgical complications and non-compliance may be exacerbated in the context of DBS treatment SE-AN, due to patients' severe chronic malnutrition (8). Moreover, the limited evidence base on efficacy and on the optimal neural target for stimulation in SE-AN makes it difficult to assess whether DBS will benefit a particular patient. Nonetheless, whilst further research is necessary, emerging evidence of DBS for SE-AN is promising (3), and our published neuroethical guidelines argue that the potential benefits of DBS can outweigh its risks and costs for some carefully selected patients (8).

Despite its attendant risks, DBS has a number of advantages as a neurosurgical intervention. First, in so far as its effects are stimulation-dependent, DBS is reversible; and on ceasing stimulation, the physical components of the DBS system can be explanted. Despite emerging evidence suggesting that DBS can lead to some non-stimulation dependent long term effects (9, 10), overall long-term evidence of other potential non-stimulation-dependent effects of DBS is lacking (11). Moreover the evidence from DBS for movement disorders suggests that significant therapeutic effects and side-effects of the procedure are stimulation-dependent. This has the important implication that patients who do not view the therapeutic effect and/or the side-effects of treatment positively can stop the effects of treatment. It is true that this will come at the cost of either a further neurosurgical procedure, or the long-term maintenance of a latent device in the body. However, we believe that the benefits of reversibility can outweigh these costs, due to the implications that it has for the control that patients may exert over their treatment. This is particularly important in the context of SE-AN, as we explain below.

As compared to lesioning not only is DBS relatively reversible, it is also patient-specific, since voltages can be fine-tuned to the specific sensitivities of the patient. Furthermore, DBS also allows for the option of exploring the effects of different neural targets. This latter feature is particularly important, given that no one neural target has proven efficacy in SE-AN. Treatment teams can modulate neural activity in a particular brain region posited to be central to a patient's pathophysiology and track neural and symptomatic effects in order to optimize and individualize the treatment. Double blinded on-off phases can also be incorporated into protocols to explore the potential influence of placebo effects (12).

However, at a time when DBS is emerging as a promising investigational treatment modality for AN, lesioning procedures in psychiatry more generally are also having a renaissance. Although ablative neurosurgery in psychiatry has a notorious history, it has been argued that technological developments and robust consent procedures have allowed for safer and more effective interventions (13).

While there is some precedent for ablative surgery in other psychiatric disorders (13), like DBS, there is currently very little evidence supporting the efficacy of ablative neurosurgery for SE-AN (14). Furthermore, there are no published systematic comparisons between DBS and ablative neurosurgery for any psychiatric indication (15). We thus currently lack the data to make an informed comparison between the effectiveness of DBS and ablative neurosurgery for SE-AN, and at this stage of experimental investigation an ablative procedure risks permanent harm.

Nonetheless, notwithstanding any potential differences in effectiveness, it has been suggested that ablative neurosurgery may have some advantages over DBS as a general treatment method for some psychiatric patients, particularly those exhibiting co-morbid substance abuse or personality disorders, even though it is irreversible, non-adjustable, and arguably poses a greater risk of neurological side-effects than DBS (15, 16). The main reason offered in favor of this view is that ablative neurosurgery does not require the long-term maintenance of an implanted device; as such, it poses lower risk of post-operative infection, and is likely to be less expensive than long-term DBS treatment (15). It might also be suggested that DBS for psychiatric disorders typically requires the use of high voltage stimulation, which might lead to short battery life and the need for major recovery surgery every several years. However, this additional concern can be circumvented by the use of rechargeable devices, which will last over 10 years even at high voltages needed, as employed in our recent trial protocol (12).

Given the considerations outlined above, some commentators have argued that DBS and ablative neurosurgery should be understood as rivaling, yet “mutually enriching paradigms” in psychiatry (16). On this “rival paradigms” approach, ablative neurosurgery is understood to be based on a “quick fix” paradigm, whilst DBS is understood to be based on a paradigm of “adjustability” (16). Neither paradigm is understood to be absolutely superior, but rather each paradigm is associated with a different set of costs and benefits for particular patients (16). On the contrary though, we believe that DBS and ablative neurosurgery are not equal rivals in the context of SE-AN. We argue that DBS should be prioritized as an investigational treatment modality for SE-AN, and that lesioning procedures are not ethically justifiable at this stage of knowledge, given the greater risk of major harms they pose, in addition to the lack of evidence regarding their efficacy.

In our view, and that of our patients, lesioning is particularly problematic for patients for whom control is central, and it potentially poses a greater risk of major harms. This claim may seem surprising, since it might be argued that ablative procedures would be practically or medically preferable to DBS for SE-AN patients in particular, due to their pronounced infection risk, and lower likelihood of compliance with long-term follow up. However, the justification for this claim is that reversibility and adjustability are particularly valuable in the context of SE-AN, because of the particular vulnerabilities of these patients (8).

SE-AN is often an ego-syntonic disorder, and patients can experience considerable ambivalence about recovery (8). With a highly limited evidence base for effective treatments, many with SE-AN have few options remaining to them and feel hopeless. At the same time, the sense of control and identity which SE-AN can provide can mean that while desperate for recovery, patients may at the same time feel terrified of losing control of the recovery process, and uncertain of their identity should they begin to recover. Psychological control thus plays a central role in the etiology and maintenance of SE-AN.

Accordingly, the adjustability and reversibility of DBS takes on a particularly significant value in SE-AN, since these features allow the individual to retain an important sphere of control, even while making a momentous decision to undergo neurosurgery. Most obviously, the patient can choose to cease stimulation, and even have the device explanted. Significantly, the patient who chooses to continue stimulation still continues to make active choices in collaboration with their treatment teams about her treatment, which reduces the likelihood of experiencing a loss of control. This adjustability also enables treatment teams to make greater allowances for the potential ambivalence of the patient toward treatment and to maintain engagement in a collaborative process of working toward recovery with DBS. In contrast, irreversible ablative neurosurgery, particularly give the lack of proven efficacy, does not allow for this sphere of control.

Indeed, patients in our current trial of DBS have echoed this line of argument. The following is a quotation from a patient interviewed about positive effects of DBS treatment for SE-AN at 12-month follow-up. The patient offered the following (unprompted) reflections on hearing about brain lesioning procedures for SE-AN:

…And I thought to myself, I wouldn't do that.. .because then it's very permanent and irreversible… although you wouldn'thave to have the device (and for a moment I thought, well, it would be nice not to have a snake running down my front,) but then I thought, no, I wouldn't want that. It seems to take the choice out of it….

Furthermore, in a recently published case study of ablative neurosurgery performed on an ego-dystonic SE-AN patient, the authors note that when the patient was asked at a 3-month follow-up whether “she would have had the surgery knowing what she knows now,” she answered negatively (17). This is despite the fact that the patient had consented to the procedure, and her condition had improved according to many objective measures. Although the patient changed to answer the question positively at 1-year follow up, this example shows the potential difficulties when deploying an irreversible procedure in patients who may experience ambivalence about their condition, and the dangers of offering irreversible surgical treatment to this patient group.

In summary, surgical ablation is a process where the alteration is “done to” the passive patient; whereas DBS is a dynamic process in which the patient is actively involved in making on-going decisions with the surgical and psychiatric team. We believe that there is a legitimate concern regarding the ethical basis, acceptability, and the potential impact of ablative neurosurgery on SE-AN patients, and DBS should be favored despite its higher cost because of its adjustability and reversibility.

## Ethics statement

This research has been granted ethics approval: Oxford A REC 13/0267 with fully informed consent from participants which includes consent for the use of anonymized quotations from their interviews within research publications.

## Author contributions

JP, JT, TA, and RP were all involved in the initial conceptual design of the paper. JP developed an initial draft. JT, TA, and RP each collaboratively reviewed and edited the manuscript for intellectual content.

### Conflict of interest statement

TA is a paid consultant for Boston Scientific, Medtronic and St. Jude Medical. He has received honoraria from Abbott, Boston and Medtronics and served as consultant to all three. The remaining authors declare that the research was conducted in the absence of any commercial or financial relationships that could be construed as a potential conflict of interest.
